# *Onchocerca* - infected cattle produce strong antibody responses to excretory-secretory proteins released from adult male *Onchocerca ochengi* worms

**DOI:** 10.1186/s12879-018-3109-6

**Published:** 2018-05-02

**Authors:** Djafsia Boursou, Dieudonné Ndjonka, Albert Eisenbarth, Kingsley Manchang, Archille Paguem, Nancy Ngwafu Ngwasiri, Jacqueline Dikti Vildina, Babette Abanda, Ralf Krumkamp, Silke van Hoorn, Alfons Renz, Mbunkah Daniel Achukwi, Eva Liebau, Norbert W. Brattig

**Affiliations:** 1 University of Ngaoundéré, Faculty of Science, Ngaoundéré, Cameroon; 2 Programme Onchocercoses, Field research station of the University of Tübingen, Ngaoundéré, Cameroon; 3 Eberhard Karls University, Institute of Evolution and Ecology, Comparative Zoology, Tübingen, Germany; 4 Veterinary Research Laboratory, IRAD Wakwa Regional Centre, Ngaoundéré, Cameroon; 5 Bernhard Nocht Institute of Tropical Medicine, Disease Epidemiology Department, Hamburg, Germany; 6 University of Münster, Münster, Germany; 7 TOZARD Research Laboratory, P.O. box 59, Bambili-Tubah, Bamenda, Cameroon

**Keywords:** *O. ochengi*, Excretory/secretory products, Onchocerciasis, IgG reactivity, Antigenicity

## Abstract

**Background:**

The front line molecules from filarial worms and other nematodes or helminthes are their Excretory-Secretory (ES) products. Their interaction with the host cells, proteins and immune system accounts for the skin and eye pathology or hyposensitivity observed in human onchocerciasis. ES products and adult worms’ crude extracts from *Onchocerca ochengi*, a filarial nematode that infects the African zebu cattle, were utilized in the present study as a model for studying *Onchocerca volvulus* that causes river blindness in man.

**Methods:**

The ES products were generated from adult male and female worms in vitro and analyzed with poly acrylamide gel electrophoresis (PAGE) and enzyme-linked immunosorbent assay (ELISA) using sera from *Onchocerca*-infected cattle and humans. The cattle sera were collected from a herd that had been exposed for six years to natural transmission of *Onchocerca* spp. The expressed reactivity was evaluated and differences analyzed statistically using Kruskal-Wallis rank and Chi-square tests.

**Results:**

The gel electrophoretic analyses of 156 ES products from *O. ochengi* female and male worms and of two somatic extracts from three females and 25 males revealed differences in the protein pattern showing pronounced bands at 15, 30–50 and 75 kDa for male ES proteins and 15, 25 and 40–75 kDa for somatic extracts, respectively and less than 100 kDa for female worms. Proteins in the ES products and somatic extracts from female and male *Onchocerca ochengi* worms were recognized by IgG in sera from both *Onchocerca*-exposed cattle and humans. Bovine serum antibodies reacted more strongly with proteins in the somatic extracts than with those in the ES products. Interestingly, the reaction was higher with male ES products than with ES products from female worms, suggesting that the males which migrate from one nodule to another are more exposed to the host immune system than the females which remain encapsulated in intradermal nodules.

**Conclusions:**

This study demonstrates that *O. ochengi* ES products and, in particular, extracts from male filariae may represent a good source of immunogenic proteins and potential vaccine candidates.

## Background

Onchocerciasis, commonly known as river blindness, remains a health problem for countries in tropical Africa, Latin America and the Arabian Peninsula. This filarial disease is caused by *Onchocerca volvulus* and nowadays affects 15.5 million people worldwide [1]. *O. volvulus* adult females live in subcutaneous nodules for more than 10 years and produce up to 1500 microfilariae per day, which can live in the skin for up to several months (or even years) whilst waiting to be taken up during a blood meal of the vector fly [2, 3].

*Onchocerca ochengi*, a bovine parasite of Zebu cattle in Africa is the closest related species to *O. volvulus* and both parasites are transmitted by the same blackfly *Simulium damnosum* sensu *lato* which breeds in fast flowing rivers, making it an appropriate natural model for studying the biology [4], chemotherapy [5] and immunology of nodule-forming *Onchocerca* parasites [6–8].

Like all helminths, nematodes are known to release products that enable them to invade, develop and persist inside a host by modulating the host immune response [9–11]. Intra and interspecific regulatory mechanisms of the worm-population are based on such putative ES-products which may also be used by the sedentary female worms to attract male worms for reproduction. Such molecules can be collected in worm cultures as in-vitro excretory-secretory (ES) products [12]. Due to their close relationship, *O. ochengi* possesses a high number of proteins homologous to those of the human parasite *O. volvulus* as evidenced on SDS-gels [13, 14]. Their closeness with regards to the elicitation of immune mechanisms was also proven via the successful immunization of cattle against *O. ochengi* using live third-stage larvae (L3) of the human parasite *O. volvulus* [15].

*O. ochengi* ES products released into the nodule fluid and produced by male adult worms during reproduction have been reported to contain proteins which may be potential vaccine candidates [16, 17]. The observed diversity noticed in this previous study was evaluated for the different developmental stages and gave an insight on the huge diversity of *O. ochengi* proteins. In addition, antigens in the somatic extract from adult female worms of *O. ochengi* were found to be as sensitive and specific as *O. volvulus* female antigens used for the diagnosis of human onchocerciasis [18].

During the course of a helminth infection, the host-parasite relationship must shift from the naïve, receptive stage, which allows the establishment of a number of worms, sufficient for reproduction and for maintaining endemicity, to a phase of ‘premunition’. This phase regulates the size of the surviving adult worm population and leads to a phase of ‘post-patency’, where worm loads may decline, while the host usually remains open to re-infection [19].

The aim of our longitudinal follow-up of a cattle herd, exposed to natural infection is the study of such shifting host-parasite relationship over the lifespan of the parasite and its host, which is in the magnitude of many years, whilst an acute immune reaction to a particular ‘antigen’ takes only two weeks to manifest. The purpose of the present work was to evaluate and compare secreted or/and excreted (ES) and extracted (Somatic Extract) proteins from female and male adult worms of *O. ochengi,* and to examine the immune recognition of these proteins by antibodies from human hosts and cattle exposed to infection during the initial phase of infection of up to 36 months.

## Methods

### *Generation of ES products:* culture of adult *O. ochengi* worms, harvesting and analysis of ES products by gel electrophoresis

Skin samples from the inguinal area of mostly female slaughtered cattle containing palpable *O. ochengi* nodules were collected as described by Wahl et al. [4] from the slaughter house of Ngaoundéré (Cameroon). Samples were taken directly to the Programme Onchocercoses field laboratory of the University of Tübingen in Ngaoundéré within two hours. Skin samples were then washed thoroughly with distilled water, then with 70% ethanol, and left to dry for 10 min. If not stated otherwise, all chemicals were purchased from Sigma-Aldrich (Deisenhofen, Germany).

Individual nodules were dissected and isolated from the skin using a scalpel blade, and put directly in phosphate buffered saline (PBS-pH 7.2). Females were isolated by digestion of the nodule with 5% collagenase for 10–15 h at 37 °C [20] and cleaned using sterile PBS supplemented with 100 U/ml penicillin and 100 μg/ml streptomycin. Male *O. ochengi* were collected by dissection of undigested nodules under a binocular microscope. Isolated males were washed three times in sterile PBS, and both, male and female worms were washed twice in the RPMI 1640 culture medium supplemented with penicillin 100 U/ml, streptomycin 100 μg/ml, L-glutamine and 0.08 mg/ml NaHCO_3_, as described by Cho-Ngwa et al. [21] and modified by Ndjonka et al. [22]. The integrity of the cultured worms was carefully checked prior to incubation. Wounded worms were generally discarded. In vitro- culture was performed in 24-well culture plates at 37 °C and 5% CO_2_ with a maximum of 25 males or, depending on the worm size, with 1–3 females per well in 2 ml of supplemented RPMI 1640 medium. Females were about 10 times longer than males. After every 48 h, culture media were changed and stored at − 20 °C in 10% trichloroacetic acid (TCA) for subsequent analysis. The culture was continued until a maximal time of 8 days, beyond which the worms were usually not alive. Dead worms were removed when detected. From the ES products produced by the research group, 250 batches were investigated in the present study.

Stored culture media were thawed in the helminthology laboratory at the Bernhard Nocht Institute for Tropical Medicine in Hamburg (BNITM) and centrifuged in 2 ml Eppendorf tubes at high speed (5000 x g for 15 min). This procedure was repeated up to six times to concentrate ES products from 3 to 6 females and up to 60 males. The supernatant was discarded, the pellet resuspended in 8 μl RPMI 1640 medium into which were added 2 μl of 10 x concentrated loading buffer (Biorad, California, USA) and heated at 95 °C for 5 min. Samples were then put on ice for 2 min, spun down (quick spin), loaded onto 10% sodium dodecyl sulphate polyacrylamide gels and, after electrophoresis, stained with 0.1% Coomassie brilliant blue stain (Carl Roth, Karlsruhe, Germany). The destaining procedure was performed with acetic acid-methanol solution and the gel was washed using distilled water for a minimum of 4 h until complete destaining of the protein-free part of the gel. The stained gel was scanned using a Canon scanner (Canon CanoScan LIDE 220; Canon, Krefeld, Germany).

### Preparation of adult worm somatic extracts

Male worms were isolated without collagenase-digestion, while female *O. ochengi* worms isolated and recovered from collagenase-digested nodules as described above, were frozen and sent to the BNITM. *O. volvulus* female worms from Ghanaian patients were provided by N. Brattig from the BNITM [23, 24]. In Hamburg, worms were thawed at room temperature (25°- 30 °C). 0.5 g and 3 g of adult male and female worms respectively were mixed with a small volume of liquid nitrogen and ground using a mortar [23, 25, 26]. Three to six milliliters of PBS was added to the ground paste, the mixture was sonicated on ice in a 15 ml Falcon tube and kept at − 70 °C for 24 h. After defrosting, the mixture was sonicated once more on ice and centrifuged at 10,000 g for 10 min. The supernatant was collected and the amount of protein evaluated using the Bradford quantification method. For the present study one batch, comprising filariae from five nodules from Ghanaian onchocerciasis patients, was applied and five batches of *O. ochengi* nodules from exposed cattle for the isolation of non-gravid female and male worms. Microfilariae were excluded. For the gel electrophoretic analysis one batch of *O. ochengi* female and male worms and females from *O. volvulus* extracts was applied. Concerning the serological analyses, ELISA tests were performed in parallel when transitioning between antigen batches and comparability between them was verified.

### Collection of sera

Sera originated from Gudali (*Bos indicus*) cattle exposed since birth to *O. ochengi* transmission for 4 months (*n* = 28) or 36 months (*n* = 24) in a paddock situated along the banks of the river Vina du Sud. The dams which produced those calves are known to be infested with variable parasite burden of *O. ochengi*. The sampled calves showed variable nodule and microfilariae loads. The nodule burden ranked form 1 to 350/cattle (median: 47) and 11 microfilariae/mg of skin were counted at 36 month post-infection of the calves. Human sera originated from the Ghanaian onchocerciasis patients exhibiting microfilarial loads of up to 106 microfilariae/mg of skin.

### Enzyme linked immunosorbent assay (ELISA)

A semi-quantitative analysis of serum IgG antibody levels was performed by ELISA to find antibody endpoints, as described previously by Mpagi et al. [27] with modifications adapted to the analysis of cattle sera. As positive controls to the ES proteins, *O. volvulus* and *O. ochengi* extracts were used in the ELISA assays. ES products from female and male *O. ochengi* as well as somatic extracts of *O. ochengi* and *O. volvulus* were used as antigens. The 96-microwell polystyrene plates (Nunc, Roskilde, Denmark) were coated with 200 ng of antigen in carbonate buffer (pH 9.6) per well and incubated overnight at 4 °C. 24 h later the plate was washed 4 times with PBS + 0.05% Tween 20. After removing unbound proteins, the plate was blocked with 200 μl of 5% bovine serum albumin (BSA) per well for 2 h at 37 °C. Excess BSA was removed by washing 4 times with PBS + 0.05% Tween 20, and the plate was incubated for another 2 h at 37 °C with dilutions between 500 and 10,000 in 200 μl PBS + 0.5% BSA of either cattle sera or sera from the 36 patients with *O. volvulus* infection from an earlier study [27]. Sera from naïve, non-exposed cattle from the University of Veterinary Medicine (Hannover, Germany) and healthy Europeans were included as negative controls. The non-specifically bound antibodies were washed out with PBS + 0.05% Tween 20 and the plate was incubated for one hour at 37 °C with 100 μl of 1/1000 diluted horseradish peroxidase-conjugated goat anti-bovine IgG or goat anti-human IgG (Sigma, St. Louis, USA). The detection was performed using 100 μl of tetramethylbenzidine (TMB) substrate in the dark for 5 min and stopping the reaction with 0.02% H_2_SO_4_ at room temperature. The optical density (OD) was measured at 405 nm using an ELISA reader (Dynatech, Denkendorf, Germany).

### Data analysis

The Kruskal-Wallis test was used for intra-group comparison, followed by Dunn’s multiple test to compare the reactivity of immunoglobulins to ES and somatic extract proteins. Optical density (OD) values were transformed into index values [28, 29] using a linear regression analysis including a boundary for negative control sera (+ 3 SD) at OD = 0.150. The distributions of the measured IgG indices for the cattle and human sera were described using the median and the interquartile range (IQR). Changes among repeated IgG measurements at two time points were compared using a Wilcoxon signed-rank test for paired data In case several samples showed no detectable IgG index, the data were dichotomised (0 = non-reactivity, 1 = detectable activity) and a Chi-square test was performed to assess differences in IgG expression. Data analysis was done using STATA 14 (College Station, TX: StataCorp LP). Statistical difference at *p* < 0.05 was considered significant.

## Results

### Harvesting and analysis of ES products

Three hundred and ten worm cultures generated by adult females or males of *O. ochengi* were collected in 2 ml of culture medium of which 250 ES products could be analysed. These ES cultures included gravid or non-gravid females and cultures with up to 25 males.

All processed samples were analysed by SDS-PAGE to detect the pattern of the respective protein bands. Only 156 ES samples showed protein bands of various molecular masses.

**Fig. 1:**
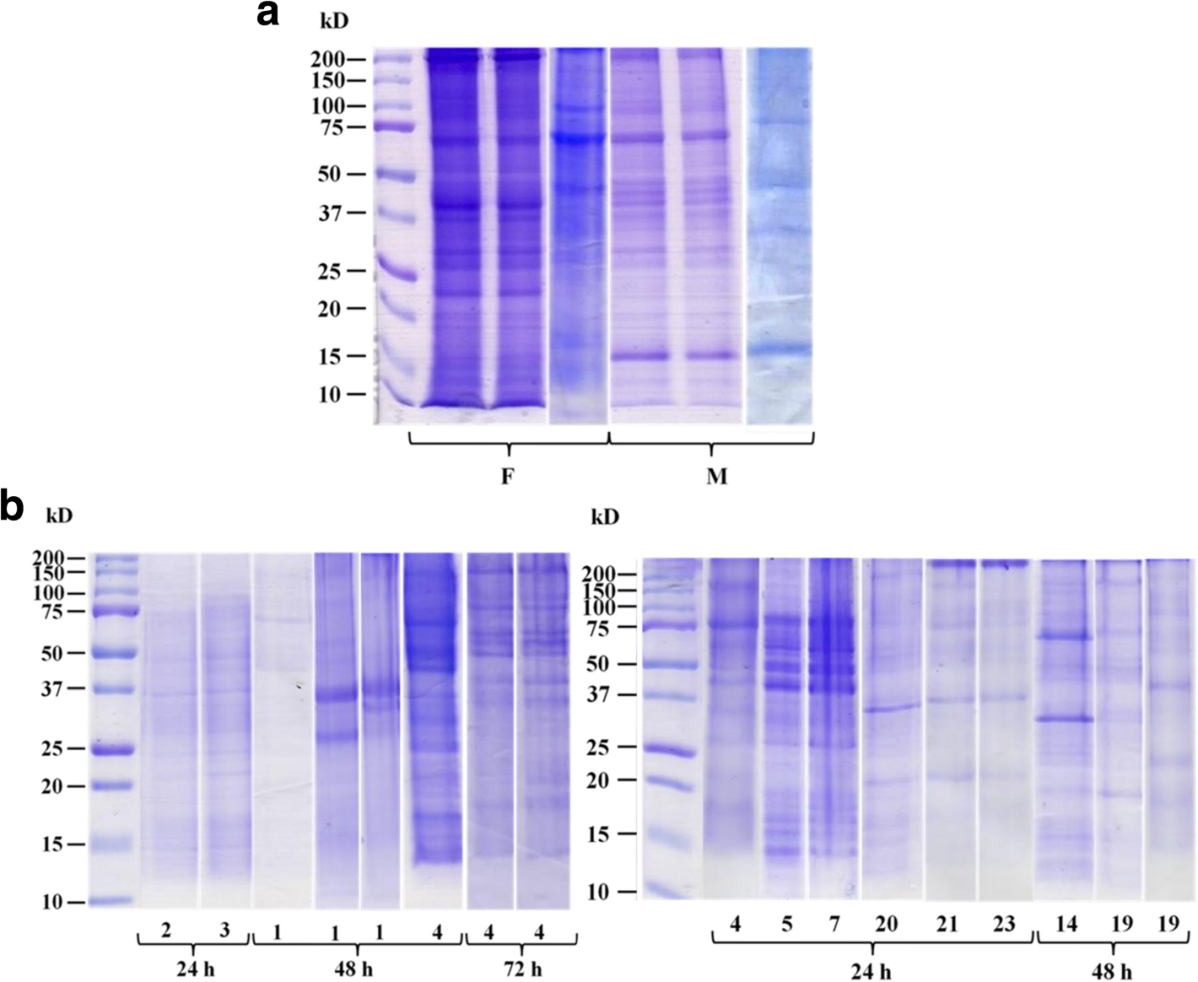
SDS-PAGE gel analysis of *O. ochengi* proteins from (**a**) somatic extracts of male (m) and female (f) worms and (**b**) of excretory/secretory (ES) products

For comparison of parasite sexes two gels were run separating extracts from non-gravid female or from male *O. ochengi* worms showing more frequent and dense proteins between 10 to > 200 kDa for female extracts (Fig. 1a). Proteins in non-gravid female extracts displayed prominent bands at 12–15, 18, 23, 27, 37–40,50, 70 and ≥ 100 kDa, while prominent protein bands in male extracts occurred at 15, 25, 30, 37–50, 100 and 150 kDa. Major differences between proteins in the two sexes included dominant bands at < 15, 23, 40 and high molecular mass > 200 kDa for female worms, while males showed a more prominent band at 15 kDa. Thus, extracts of female worms comprised different proteins from those of male worms.

In spite of the remarkable variability of the ES samples’ protein patterns, a higher proportion of ES proteins from females were of low molecular mass with protein bands between 30 and 50 and ≤ 100 kDa. Male ES proteins instead showed prominent band at 15–27 and 50–75 kDa (Fig. 1b). At 48 h, the 4 females’ ES products showed more prominent protein bands as compared to the other 23 males’ ES products protein patterns. For female ES products after 24 h, two lanes (one for products from 2 females and the other one for those from 3 females), for which the band separation and intensity are very similar, are shown (Fig. 1b).

### Immune recognition of somatic extract and ES proteins from female and male worms of O. ochengi and of somatic extract of O. volvulus female filariae by IgG in sera from bovine and human hosts

**Fig. 2:**
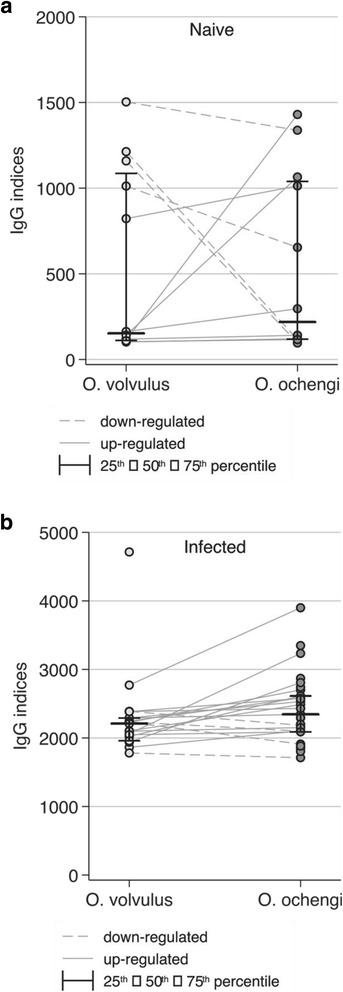
IgG recognition of proteins in the somatic extracts from *O. volvulus* and *O. ochengi* female worms using sera from non-exposed persons (a: Naïve) and onchocerciasis patients (b: Infected)

**Fig. 3:**
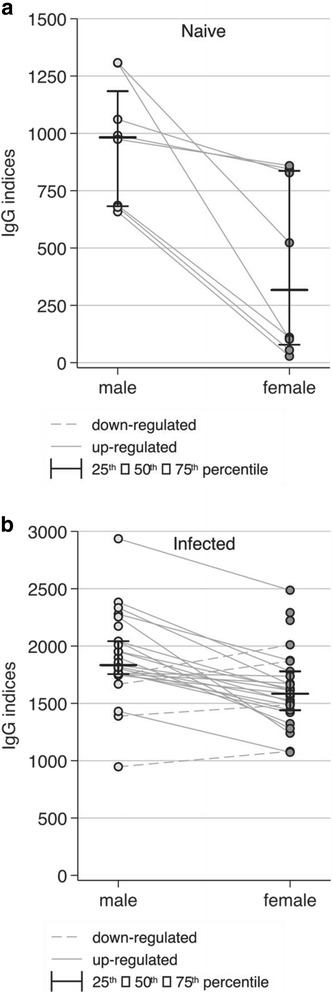
IgG recognition of proteins in ES products from *O. ochengi* females and males by sera from non-exposed persons (**a:** Naïve) and onchocerciasis patients (**b:** Infected)

Antigens (extracts and ES products) from male and female worms of both *O. volvulus* and *O. ochengi* were recognized in a similar manner by human IgG antibodies from infected patients confirming the closeness between these two species (Figs. 2 and 3).

In total, 28 cattle were included in the longitudinal analysis comparing bovine sera from animals exposed for 4 or 36 months. However, varying numbers of measurements were available at both time points. Figure 3 and Table 1 show the IgG indices for sera from cattle exposed for 4 and 36 months reacting with antigens in extracts of female and male *O. ochengi*. At the 4th month no reactivity was detected in 6 (27%) out of 22 cattle for female and for one (4%) out of 25 cattle for male antigens. All sera from cattle that had been exposed for 36 months reacted with the proteins in female extracts with an index range between 600 and 1750.

**Table 1:** Comparison of indices of antibody reactivities with antigens in female or male somatic extracts using sera from cattle taken after 4 or 36 months of exposure

Antigen extract, Exposure time	Indices of IgG reactivity
	Median (IQR)
Female extract, month 4	1068 (0–1479)
Female extract, month 36	1154 (937–1400)
Male extract, month 4	1739 (1526–1861)
Male extract, month 36	1641 (1490–1933)
	Wilcoxon signed-rank test
Female extract, month 4 vs. Female extract, month 36	*p* = 0.98
Male extract, month 4 vs. Male extract, month 36	*p* = 0.35
Female extract, month 4 vs. Male extract, month 4	*p* < 0.001
Female extract, month 36 vs. Male extract, month 36	*p* < 0.001

*IQR* interquartile range

Looking at the overall distribution of the indices representing the IgG recognition of male as opposed to female ES products and extracted somatic antigens, no significant difference between months 4 and 36 was observed. However, indices of IgG recognition for male *O. ochengi* were at both time points significantly higher than indices for female parasites (*p* < 0.001).

**Fig. 4:**
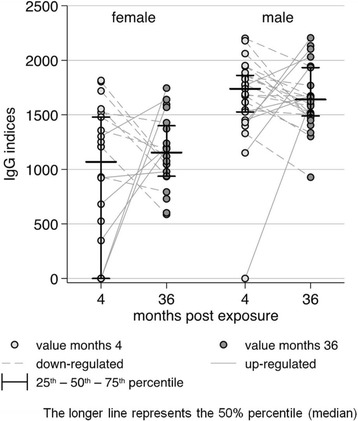
Serum IgG indices of the recognition of antigens in somatic extracts from adult female and male *O. ochengi* filariae

Repeated index measures using female extract as antigen from both time-points were available from 14 cattle, of which 6 (43%) had an increased and 8 (57%) a decreased value at month 36 (Fig. 4). Similar results were obtained using extracts from male parasites. From 23 cattle repeated IgG measurements were available, of which 7 (30%) had an increased and 16 (70%) had a decreased index in samples taken at month 36.

**Fig. 5:**
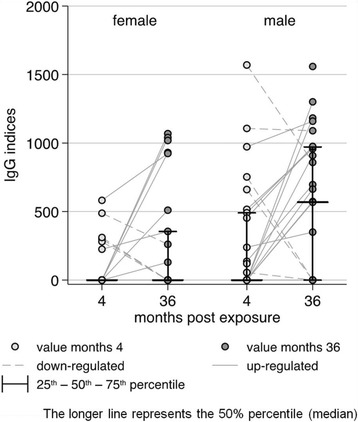
Distribution of IgG indices for 28 sera from cattle exposed for 4 and 36 months to *Onchocerca* transmission reactive with ES proteins from female and male *O. ochengi* adult worms

The IgG indices of reactivity with ES proteins from male and female worms are shown in Fig. 5 and Table 2. Measurements from 25 cattle at month 4 and from 27 cattle at month 36 for both female and male worms of *O. ochengi* were available. Nineteen (76%) cattle sera showed no reactivity to female ES proteins at months 4 (non-responders) and five (26%) of these non-responders reacted after 36 months exposure to transmission by the vector *S. damnosum*. The proportion of detectable IgG indices for ES proteins was comparable at both time points.

**Table 2:** Comparison of indices of cattle antibody reactivities with female or male excretory/secretory (ES) products after 4 or 36 months of exposure

Worm sex, Exposure time (number of worms)	Indices of IgG reactivity for ES proteins (if > 0)
	Median (IQR)
female, months 4 (6)	305 (280–488)
female, months 36 (9)	924 (355–1019)
male, months 4 (12)	504 (188–863)
male, months 36 (15)	958 (664–1160)
	Indices for ES proteins = 0 vs. ES proteins > 0
	Chi square test
female 4 vs. female 36:	6/25 (24%) vs. 9/27 (33%); *p* = 0.46
male 4 vs. male 36:	12/25 (48%) vs. 15/27 (56%); *p* = 0.59
female 4 vs. male 4:	6/25 (24%) vs. 12/25 (48%); *p* = 0.08
female 36 vs. male 36:	9/27 (33%) vs. 15/27 (56%); *p* = 0.10

*IQR* interquartile range

Repeated measures for female ES proteins were available for 24 cattle. In the latter, the recognition of the ES proteins remained unchanged (at a value of 0) at both time-points in 13 (54%), increased in 7 (29%) and decreased in 4 (17%) cattle. The proportion of samples with increased/decreased IgG indices for ES proteins at month 4 and month 36 was too small for further statistical evaluation. Absence of antibody recognition of ES proteins from male parasites (non-responders) occurred in 13 cattle (52%) at months 4 and in 12 (44%) cattle at month 36 indicating no difference between both observation time points. Seven cattle did not react with the male ES proteins (non-responders) at both exposure times. Increased IgG indices were observed in 12 (50%) while decreased indices occurred in 5 (21%) cattle. The number of repeated increased values for the ES proteins from female parasites was also too low for further evaluation.

Comparing IgG indices at months 4 and 36 indicated that ES proteins from males were more often recognized than female ES antigenic proteins. There was, however, no significant difference for this observation (*p* = 0.08 and *p* = 0.10, respectively). In the first time point (4 months) sera from 6 cattle (24%) showed measurable index values for female antigens and 12 (48%) for antigens in male ES while at the second time point (36 months) 9 (33.3%) sera reacted with female and 15 (55.6%) with male ES proteins (Table 2).

## Discussion

Proteins released by filarial worms play a significant role in the interaction with their hosts and on the filarial survival as well [30, 31]. In the present study, *O. ochengi* ES products collected from female and male worms after various time points revealed diversity in the proteins related to the time of culture and to the number of cultured worms (Fig. 1). At a given time and for a similar number of worms, the displayed band patterns were not absolutely the same, therefore reflecting some possible inner variability of the worms ES products. Elsewhere, a time-dependent protein release was reported for nematodes [32–34]. On notice, the dead or wounded worms with fluid leaking from their body wall were discarded. Any effect of the digestion of the nodule on the vitality and integrity of the females may be excluded since during the first 24 h of culture, only marginal protein release from females was observed which increased during the following 48 h (Fig. 1b).

Regarding recognition of ES proteins by cattle sera, it is striking that the indices tended to increase with time in animals which had low indices at 4 months, but fell, when the indices were already high at this young age. There must be an optimum (or undulating) distribution for the indices of individual animals, with a maximum at any time-point before, in between or after 4 to 36 months. More detailed studies on the longitudinal dynamics of the IgG levels over the full course of infection (more than 6 years) and at higher frequencies (bi- or three-monthly intervals) are still under course.

For *O. volvulus*, partially similar protein bands had been observed by Sakwe et al. [33] as at 10–20 kDa, about 40 kDa and 60 kDa*.* Cho-Ngwa et al. [35] analyzing nodular fluid proteins, so-called in vivo ES products of *O. ochengi*, observed partially similar protein bands. Thus, like Cho-Ngwa et al. (2011), in the present study we also found prominent protein bands between 40 and 70 and 90 kDa, in particular for ES from male filariae (Fig. 1b) which may comprise the dominant protein OV1CF of 62.4 kDa reported by Cho-Ngwa et al. [35]. This indicates that these proteins may be released from living males or females in the nodule. In addition, the 41 kDa enolase and 38 kDa glyceraldehyde-3-phosphate-dehydrogenase listed by Cho-Ngwa et al. may be present in these ES proteins which were studied by our group [36, 37]. Three generated potential diagnostic monoclonal antibodies also reacted with proteins of 20–220 kDa which were also recognized by sera from *Onchocerca*-infected cattle as well as humans [21]. The similar reactivity of the sera from *O. ochengi*-infected cattle to that of *O. volvulus*-infected humans with both, *O. volvulus* and *O. ochengi* proteins reflects the reported physiological closeness [13] and phylogenetic relationship between the two parasites [16, 38].

The present study is limited to a description of a demonstration of multiple differing proteins in male and female ES products. More detailed analysis can be addressed by mass spectrometry analyses [14].

No correlation between the indices for the IgG recognition of antigens in the extracts and ES proteins measured at months 4 were observed. However, positive ES indices measured at months 36 were mainly detected in cattle with increased indices for recognition of extract antigens.

The present results depict the diverse pattern of immune reactivity of cattle sera with proteins arising from male and female worms. The higher antibody response to male ES proteins can be related to their migratory behaviour in the host as compared to the sessile females in the nodules leading to increased immune interference. This result has not yet been published from any other filaria or helminth. Of note, cattle IgG1 is reported to be transferred maternally through colostrum [25], which can be detected in the investigated sera and which can operate in the calves during the first months and possibly may induce the reduced establishment of *O. ochengi* in the calves.

The decreased reactivity observed in many infected cattle could refer to the modulation of the immune system by the parasite which may be mediated by released products. An important impact on the immunological reactivities may result from the frequent co-infection by filarial species or other nematodes in our cattle e.g. by *O. gutturosa, O. armillata* and *Setaria* etc. [39]. In addition, some contributions may be attributed to compounds of the endosymbiotic *Wolbachia* endobacteria, present in female as compared to male filariae [24, 26, 40–42].

Parasitic helminths secrete products which have been increasingly reported to dampen their host immune response against them. These products referred to as immunomodulatory components of helminths facilitate the establishment and propagation of the parasite [9–11, 30, 43]. Further investigations namely mass spectrometry and bioinformatics analyses on these *Onchocerca* ES products are currently being performed [14, 16]. In order to elaborate an efficient vaccine against onchocerciasis, identifying the proteins in *O. ochengi* ES products and analysing their structure and functions in host-parasite interactions should definitely be the main focus.

## Conclusions

The performed ELISA analyses demonstrated the antigenicity of *Onchocerca spp.* proteins. Furthermore, the observed cross-reactivity is an extra proof of the phylogenetic closeness of *O. volvulus* and *O. ochengi*. More specifically, this study firstly revealed a stronger recognition of male filariae proteins by host antibodies. The detection, isolation and hybridization of each involved *O. ochengi* ES protein will create the possibility to evaluate their potentials as vaccine candidates.

## Abbreviations

BSA: Bovine serum albumin
ELISA: Enzyme linked immuo-sorbent assay
ES: Excretory and secretory
IQR: Interquartile range
PBS: Phosphate buffered saline
SDS PAGE: Sodium dodecyl sulphate polyacrylamide gel electrophoresis
TCA: Trichloroacetic acid
TMB: Tetramethylbenzidine
